# Revisiting *Nesaulax* Roman (Hymenoptera, Braconidae, Braconinae), with description of a new species from China

**DOI:** 10.3897/zookeys.1267.137062

**Published:** 2026-01-28

**Authors:** Yang Li, Cornelis van Achterberg, Cheng-Jin Yan, Jia-Chen Zhu, Xue-Xin Chen

**Affiliations:** 1 Sichuan Provincial Forestry and Grassland Key Laboratory of Biodiversity Conservation and Sustainable Community Development in Giant Panda National Park, College of Chemistry and Life Sciences, Chengdu Normal University, Chengdu 611130, China Zhejiang University Hangzhou China; 2 State Key Laboratory of Rice Biology, Ministry of Agriculture Key Lab of Molecular Biology of Crop Pathogens and Insects, Zhejiang Key Laboratory of Biology and Ecological Regulation of Crop Pathogens and Insects, and Institute of Insect Sciences, Zhejiang University, Hangzhou 310058, China Zhejiang A&F University Hangzhou China; 3 Wenzhou Vocational School of Science and Technology, Wenzhou 325006, China Chengdu Normal University Chengdu China; 4 College of Advanced Agricultural Sciences, Zhejiang A&F University, Hangzhou 311300, China Wenzhou Vocational School of Science and Technology Wenzhou China

**Keywords:** Aphrastobraconini, braconine wasps, identification key, new record, new species, new synonym, Oriental

## Abstract

The genus *Nesaulax* Roman, 1914 (Hymenoptera, Braconidae, Braconinae) is reported from China for the first time, and a new species (*Nesaulax protuberator* Li & van Achterberg, **sp. nov**.) is described and illustrated. Our pairwise genetic results indicated that four *Nesaulax* specimens from Thailand, Malaysia, and India in the BOLD system are conspecific with our newly described species. In addition, we report a new synonym: *Nesaulax gracilis* (Enderlein, 1920) **syn. nov**. of *N. pravivena* (Enderlein, 1920). A key to the species of the genus *Nesaulax* is provided for the first time.

## Introduction

*Nesaulax* Roman, 1914, is a small genus of the subfamily Braconinae (Hymenoptera, Braconidae) with eight described species worldwide (Yu et al. 2016; Quicke et al. 2023). The biology of this genus is still unknown, but related genera are ectoparasitoids of coleopterous larvae. *Nesaulax* occurs only in the Oriental region (Yu et al. 2016). So far this genus is unknown from China, but among Braconinae in the collection of the College of Chemistry and Life Sciences of Chengdu Normal University (Chengdu), a new species from Hainan (SE China) was discovered. In the present paper, the new species is described and illustrated, and a new key to the species of the genus *Nesaulax* is provided.

## Material and methods

The examined specimen was collected using a Malaise trap. Monthly collected specimens from Malaise traps (located in Hainan, southern China) were kept in 100% ethanol and mounted on point cards or with #3 insect pins. The holotype specimen is deposited in the College of Chemistry and Life Sciences, Chengdu Normal University, Chengdu (**CDNU**).

The terminology and measurements used follow van Achterberg (1988, 1993). The medial length of the third metasomal tergite is measured from the posterior border of the second suture to the posterior margin of the tergite.

Photographs were made with a Canon 6D Mark II digital camera with a Laowa 25 mm f2.8 + 2.5-5.0 × lens, and those of the apex of the antenna and ovipositor with a Mitutoyo 10x lens. The photos were slightly processed (mainly cropped and the background modified) in Photoshop 2024. For the descriptions and measurements, an Olympus SZX7 stereomicroscope was used.

Genomic DNA was extracted from the left middle leg of the specimen using a DNeasy Blood & Tissue Kit (QIAGEN, Inc.), following a non-destructive DNA extraction protocol as described in Taekul et al. (2014). Following DNA extraction, the “barcode” region of the mitochondrial cytochrome *c* oxidase subunit 1 (COI) was amplified using the LCO1490/HCO2198 (Folmer et al. 1994) primer pair. Polymerase chain reactions (PCRs) were performed using Tks Gflex DNA Polymerase (Takara) and conducted in a T100 Thermal Cycler (Bio-Rad). Thermocycling conditions were: an initial denaturing step at 94 °C for 4 min, followed by 30 cycles of 94 °C for 40 s, 50 °C for 40 s, 72 °C for 45s, and an additional extension at 72 °C for 5 min. Amplicons were directly sequenced in both directions with forward and reverse primers on an Applied Biosystems (ABI) 3730XL by Guangzhou Tianyi Huiyuan Gene Technology Co., Ltd. (Guangzhou, China). The residual DNA is archived (–30 °C) in the molecular laboratory of SCBG, Guangzhou, China, and is available for further study upon request.

The forward and reverse sequencing results were assembled and edited in Geneious ver. 2023.2.1. We used BLAST in NCBI database to preliminarily verify the reliability of the sequences. After curation, the sequence was submitted to NCBI (GenBank accession number: PV546357) and BOLD (accession number: NESAU001-25). We also downloaded all BINs sequences of *Nesaulax* in Barcode of Life Data (BOLD, https://boldsystems.org/ (accessed on 21 October 2024) platform for analysis.

## Results

The K2P genetic distances calculated from COI sequences revealed low levels of genetic differentiation among the *Nesaulax* specimens examined (Table 1). *Nesaulax protuberator* sp. nov. exhibited minimal genetic divergence from other *Nesaulax* specimens. The K2P distances ranged from 0.0020 (99.80% similarity) with *Nesaulax* sp. NML-2000 AJ231541 (Malaysia) to 0.0112 (98.88% similarity) with *Nesaulax* sp. CCDB-06325-C09 (Thailand) and CCDB-06326-D09 (India). The genetic distance to *Nesaulax* sp. CCDB-32188-G09 (Thailand, approximately 200 bp) was intermediate at 0.0052 (99.48% similarity). The low genetic distances observed (all < 1.2%) indicate that the COI gene is highly conserved within this group.

**Table 1.** The interspecies K2P genetic distance ranges for COI sequences.

	*Nesaulax* sp. CCDB-06325-C09 (BBTH2595-21)	*Nesaulax* sp. CCDB-06326-D09 (BBTH2702-21)	*Nesaulax protuberator* sp. nov.	*Nesaulax* sp. CCDB-32188-G09
*Nesaulax* sp. CCDB-06326-D09 (BBTH2702-21)	0 (100%)	/	/	/
*Nesaulax protuberator* sp. nov.	0.0112 (98.88%)	0.0112 (98.88%)	/	/
*Nesaulax* sp. CCDB-32188-G09	0.0156 (98.44%)	0.0156 (98.44%)	0.0052 (99.48%)	/
*Nesaulax* sp. NML-2000 AJ231541 (GBAHB1498-18)	0.0123 (98.77%)	0.0123 (98.77%)	0.0020 (99.80%)	0.0052 (99.48%)

Note: The number in parentheses is the process ID.

These results strongly suggest that *Nesaulax protuberator* sp. nov., the *Nesaulax* sp. NML-2000 AJ231541 (Malaysia), and *Nesaulax* sp. CCDB-06325-C09 (Thailand) and CCDB-06326-D09 (India) are conspecific. Later examination of the Thai specimen with the largest genetic distance (Table 1) by photographs showed no morphological difference worth to consider it a separate species, therefore, the sequenced specimens listed in Table 1 are considered to be conspecific.

The key is based on examination of all types, either physically (holotypes of *N. flagellaris* and *N. protuberator*) or by photos kindly supplied by Konstantin Samartsev and Chengjin Yan. According to this survey, our analysis confirms that the morphological characteristics of the specimens at hand are not consistent with any known species, and, therefore, we formally describe this taxon as a new species.

### Key to species of *Nesaulax* Roman

**Table:** 

1	Fourth metasomal tergite coarsely irregularly and densely rugose (Fig. 1e); fourth tergite with reversed V-shaped emarginated medio-posteriorly (Fig. 1f); length of body about 6.5 mm; [vein cu-a of fore wing nearly vertical; apical antennal segments of ♀ whitish; veins 1-SR+M and CU1 of fore wing straight; Indonesia; type species of *Antidiolcus* Enderlein, 1920]	***N. excisus* (Enderlein, 1920)**
–	Fourth metasomal tergite mainly smooth or with fine and regular (more or less curved) striae (Fig. 4e); fourth tergite truncate medio-posteriorly; length of body 8–16 mm	**2**
2	Apical antennal segments of ♀ black; basal half of pterostigma partly yellowish; fourth metasomal tergite finely and densely striate; [Sri Lanka]	***N. greeni* (Cameron, 1905)**
–	Apical segments (12–15) of antenna yellowish or whitish (unknown of *N. dehaani*); basal half of pterostigma dark brown or blackish; fourth tergite at least partly smooth	**3**
3	Vein 1-SR of fore wing oblique (Fig. 2j); hind coxa and femur yellowish brown; third tergite without crenulated subapical groove (Fig. 2g); [ovipositor sheath of holotype lost; Philippines; type species of *Nesaulax* Roman, 1914]	***N. flagellaris* (Roman, 1914)**
–	Vein 1-SR of fore wing subvertical (Fig. 1c); hind coxa and femur black, dark brown or testaceous; third tergite often with distinctly crenulated subapical transverse groove (Fig. 4e)	**4**
4	Vein cu-a of fore wing vertical or slightly reclivous (angle with vein M+CU1 about 45°), non-linear with vein 1-M (Fig. 4a); vein 1-SR+M distinctly curved (Fig. 4a); [China, Thailand, Malaysia, India]	***N. protuberator* sp. nov**.
–	Vein cu-a of fore wing strongly reclivous (angle with vein M+CU1 about 30°), linear with vein 1-M (Figs 1c, 2a); vein 1-SR+M weakly curved (Fig. 2a)	**5**
5	Vein CU1 of fore wing straight and narrow (Fig. 1c); anteriorly vein 3-CU1 distinctly angled with vein CU1; [Malaysia (Sarawak)]	***N. ornaticornis* (Cameron, 1904)**
–	Vein CU1 of fore wing distinctly curved and slightly widened (Figs 1a, b, d); anteriorly vein 3-CU1 gradually merging into vein CU1	**6**
6	Vein m-cu of fore wing 0.6 times as long as vein 1-M; vein 1-SR pointing to basal third of vein CU1; [vein 1-SR+M of fore wing distinctly curved; Indonesia]	***N. dehaani* (Cameron & Strand, 1912)**
–	Vein m-cu of fore wing 0.4 times as long as vein 1-M; vein 1-SR pointing to middle of vein CU1 (Fig. 1a)	**7**
7	Vein 1-M of fore wing slightly curved; vein m-cu of fore wing distinctly wider than vein CU1; vein cu-a of fore wing curved posteriorly; [Sri Lanka]	***N. ernesti* (Cameron, 1905)**
–	Vein 1-M of fore wing straight (Fig. 1a); vein m-cu of fore wing as wide as vein CU1 (Fig. 1a); vein cu-a of fore wing straight posteriorly; [= *N. gracilis* (Enderlein, 1920) syn. nov.; Indonesia; type species of *Plagiozina* Enderlein, 1920]	***N. pravivena* (Enderlein, 1920)**

**Figure 1.**
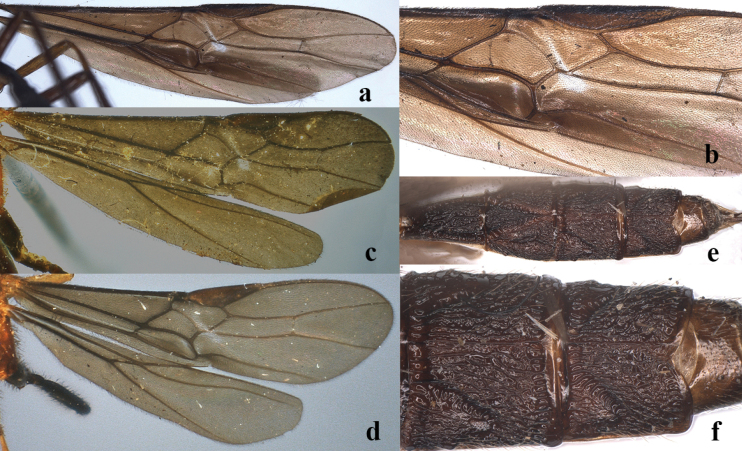
♀. **a**. *Nesaulax pravivena* (Enderlein, 1920), lectotype, fore wing; **b**. *Nesaulax pravivena* (Enderlein, 1920), lectotype, part of fore wing; **c**. *Nesaulax ornaticornis* (Cameron, 1904), holotype, wings; **d**. *Nesaulax ernesti* (Cameron, 1905), holotype, wings; **e**. *Nesaulax excisus* (Enderlein, 1920), lectotype, metasoma, dorsal aspect; **f**. *Nesaulax excisus* (Enderlein, 1920), lectotype, third to fourth metasomal tergites, dorsal aspect.

### Taxonomy

#### Nesaulax

Taxon classificationAnimaliaHymenopteraBraconidae

Roman, 1914

3D9CDFBA-0FBB-51D1-9AE3-FA23CBDAD904

Figs 3, 4

Nesaulax Roman, 1914: 2. Type species: *lpobracon* (Nesaulax) flagellaris Roman, 1914.
Antidiolcus Enderlein, 1920: 95. Type species: Antidiolcus excisus Enderlein, 1920. Synonymized by Quicke 1984: 44.
Plagiozina Enderlein, 1920: 94. Type species: Plagiozina pravivena Enderlein, 1920. Synonymized by Quicke 1984: 44.

##### Diagnosis.

**Figure 2.**
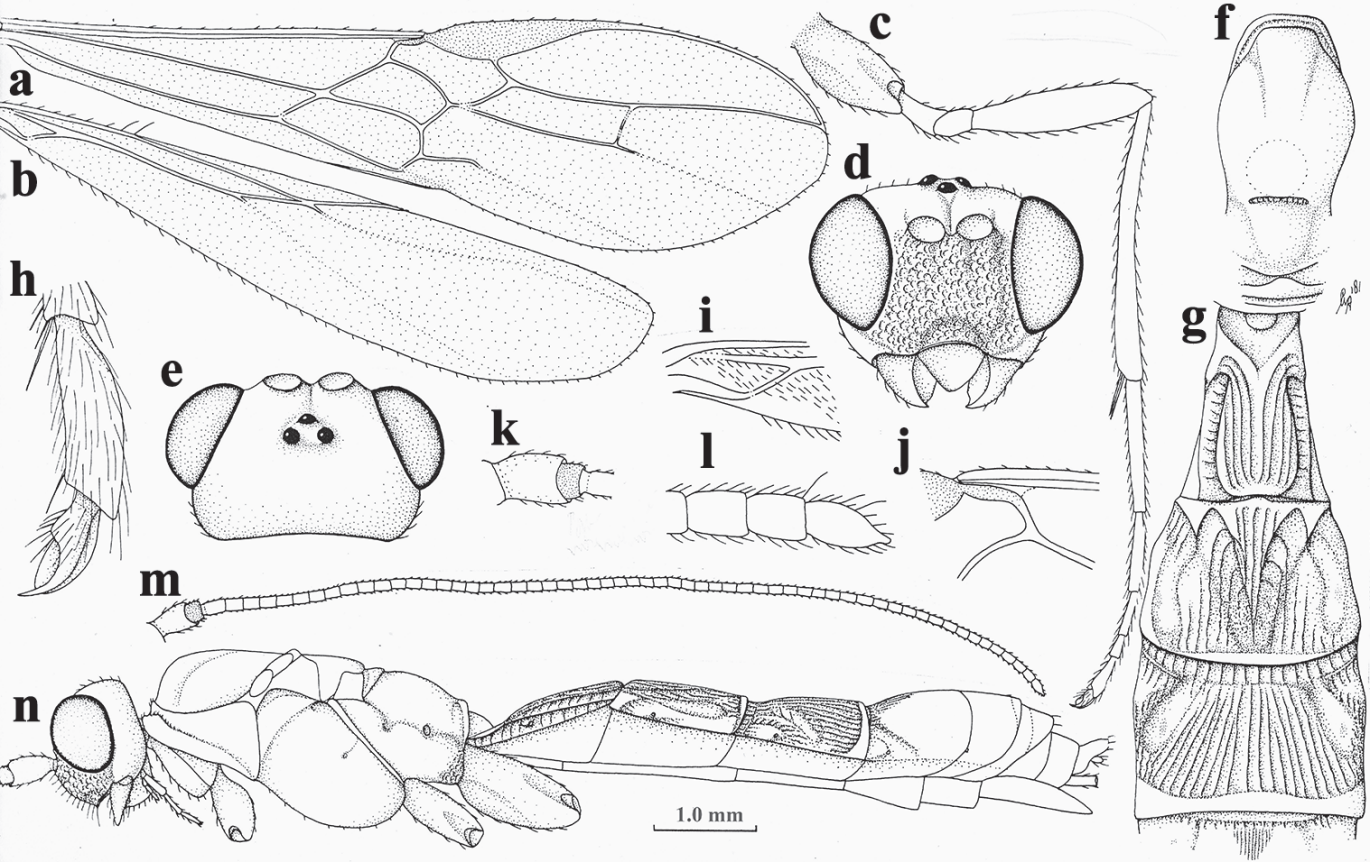
*Nesaulax flagellaris* (Roman, 1914), ♀, holotype. **a**. Fore wing; **b**. Hind wing; **c**. Hind leg, lateral aspect; **d**. Head, anterior aspect; **e**. Head, dorsal aspect; **f**. Mesosoma, dorsal aspect; **g**. First to third metasomal tergites, dorsal aspect; **h**. Apex of hind tarsus, lateral aspect; **i**. Basal part of hind wing; **j**. Fore wing vein 1-SR; **k**. Scapus outer side, lateral aspect; **l**. Apex of antenna; **m**. Antenna; **n**. Habitus, lateral aspect.

**Figure 3.**
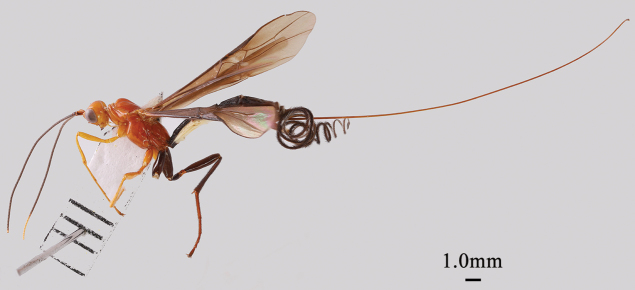
*Nesaulax protuberator* Li & van Achterberg, sp. nov., ♀, holotype, habitus, lateral aspect.

**Figure 4.**
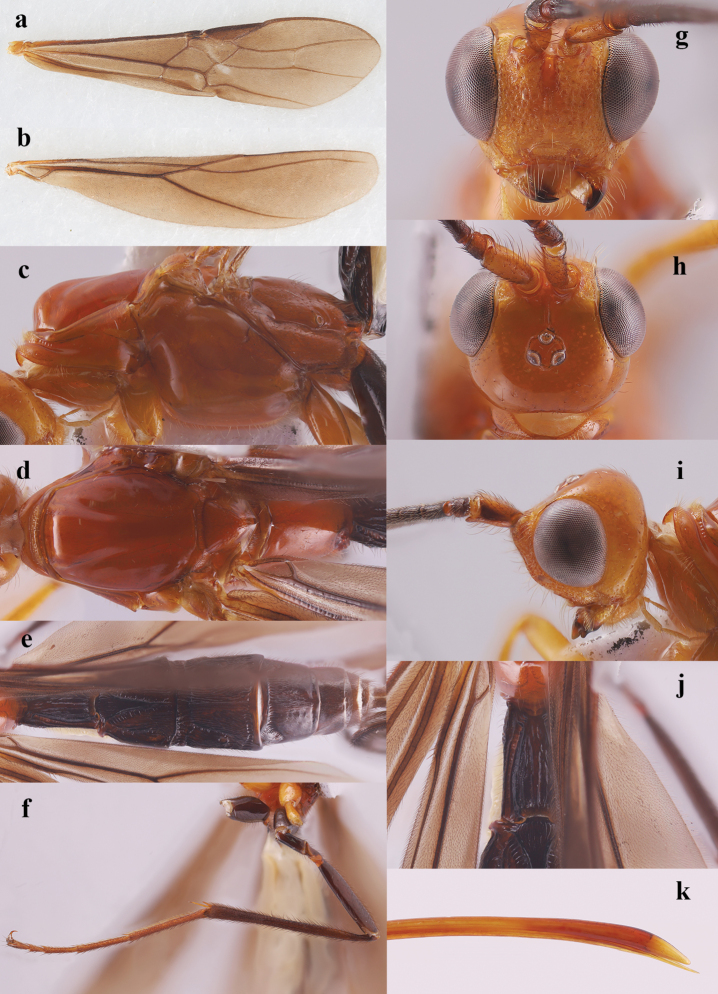
*Nesaulax protuberator* Li & van Achterberg, sp. nov., ♀, holotype. **a**. Fore wing; **b**. Hind wing; **c**. Mesosoma, lateral aspect; **d**. Mesosoma, dorsal aspect; **e**. Metasoma, dorsal aspect; **f**. Hind leg, lateral aspect; **g**. Head, anterior aspect; **h**. Head, dorsal aspect; **i**. Head and scapus outer side, lateral aspect; **j**. First metasomal tergite, dorsal aspect; **k**. Apex of ovipositor, lateral aspect.

Middle flagellar segments of antenna distinctly longer than wide (Fig. 2m), with densely short setae, in lateral view scapus often protruding apico-ventrally (Fig. 4i), but in type species not protruding (Fig. 2k); eye glabrous, weakly or not emarginated (Figs 2d, 4g); face usually coarsely punctate (Fig. 4g); clypeus relatively wide; with (Fig. 4g) or without (Fig. 2d) dorsal carina; malar suture present; labio-maxillary complex normal, not elongate; frons with deep median groove, smooth (Fig. 4i); labrum concave; pronotum slightly protruding and horizontal anteriorly (Fig. 4c); mesoscutum smooth; notauli impressed on disc anteriorly, becoming rather shallow posteriorly; propleuron largely glabrous except with a row of dense long setae (Fig. 4d); mesopleuron sparsely setose posteriorly; pleural sulcus smooth; metapleural flange distinct (Fig. 4c) or absent (Fig. 2n); scutellar sulcus comparatively wide, and sparsely (Fig. 4d) or densely (Fig. 2f) crenulate; metanotum convex medially, without median carina anteriorly (Fig. 4d); propodeum smooth, at most with few short carinae latero-posteriorly, medio-longitudinal carina or groove absent (Fig. 4d); propodeum spiracles ovoid (Fig. 4c); angle between veins 1-SR and C+SC+R of fore wing about 80° (Fig. 4a) or less (Fig. 2j); pterostigma relatively slender (Fig. 4a); vein 1-M of fore wing straight; vein 1-SR+M of fore wing evenly curved; fore wing vein CU1b shorter than vein 3-CU1; fore wing second submarginal cell slender and parallel-sided apically (Fig. 4a); fore wing vein 3-SR of fore wing distinctly shorter than vein SR1 (Fig. 4a); fore wing vein 3-CU1 normal, not widened apically (Fig. 4a); fore wing vein cu-a weakly postfurcal or interstitial; hind wing vein SC+R1 rather long, vein 1r-m shorter than vein SC+R1; vein 2-SC+R of hind wing longitudinal, rather short; hind wing glabrous around vein cu-a (Fig. 4b); claws simple (Fig. 4f); fore tarsus elongate, legs more or less with dense and long setae (Fig. 3); metasomal tergites entirely sclerotized and exposed; first metasomal tergite relatively slender, about 1.4 times longer than its apical width or longer, nearly parallel-sided; median area of first tergite developed, with some long longitudinal rugae on it (Fig. 4j); second metasomal tergite coarsely sculptured, largely with longitudinal and oblique striae including large medio-basal area (Figs 2g, 4e); second suture deep and rather wide, crenulate, slightly curved upward laterally, straight medially (Fig. 4e); third-fourth metasomal tergites largely smooth or with longitudinal striae except for posterior border, and with convex antero-lateral areas and transverse subposterior groove (Figs 2g, 4e); fifth–seventh metasomal tergites smooth; hypopygium acute apically, reaching beyond level of apex of metasoma; ovipositor sheath more than 1.5 times as long as fore wing; ovipositor aberrant, its upper valve strongly enlarged and without nodus, lower valve without teeth and needle-shaped (Fig. 4k).

##### Distribution.

Oriental: China (Hainan)*; India; Indonesia; Malaysia; Philippines; Sri Lanka. This genus is new to China.

##### Notes.

*Merinotus gracilis* Enderlein, 1920 is a new synonym of *Plagiozina pravivena* Enderlein, 1920 (syn. nov.), as no differences were found after examining photographs of the types kindly provided by Konstantin Samartsev.

#### Nesaulax protuberator

Taxon classificationAnimaliaHymenopteraBraconidae

Li & van Achterberg sp. nov.

5E023ACA-3F07-5720-A2C7-057BF65C9B9D

https://zoobank.org/BBD801EE-0DE4-4E7C-B815-3BD7CCA840B2

Figs 3, 4

##### Type material.

***Holotype***. ♀, China, • Hainan Prov., Mt. Diaoluo, 18°40'3.72"N, 109°53'54.3"E, 31.X-30.XI.2020, Chen Longlong, No. HN17, LSX953, 2022001 (CDNU).

##### Diagnosis.

Details are in the key to the species of *Nesaulax* Roman, 1914.

##### Description.

Holotype, ♀, length of body 13.0 mm, of fore wing 11.0 mm, of ovipositor sheath 23.5 mm.

***Head***. Antenna incomplete, with 59 segments remaining; median segments 1.5 times longer than wide; third segment 1.3 and 1.4 times longer than fourth and fifth, respectively, the latter 1.8 times longer than wide; clypeus height: inter-tentorial distance: tentorio-ocular distance = 6: 19: 11; face coarsely punctate and sparsely setose (Fig. 4g); eye height: shortest distance between eyes: head width = 18: 17: 34; frons smooth, strongly concave behind antennal sockets (Fig. 4h); vertex smooth and with few setae; minimum distance between posterior ocelli: minimum diameter of elliptical posterior ocellus: minimum distance between posterior ocellus and eye = 1: 1: 3; in dorsal view length of eye 1.4 times temple; temples gradually narrowed behind eyes, rounded posteriorly, largely smooth and with few short setae (Fig. 4h).

***Mesosoma***. Length of mesosoma 2.1 times its height (Fig. 4c); propleuron and pronotum densely setose anteriorly (Fig. 4c); mesopleuron and metapleuron smooth and glabrous (Fig. 4c); notauli impressed on disc anteriorly, becoming rather shallow posteriorly (Fig. 4d); mesoscutum smooth and glabrous, middle lobe weakly convex (Fig. 4d); scutellar sulcus moderately wide and deep, with short crenulae (Fig. 4d); scutellum largely smooth, sparsely punctate and setose posteriorly; metanotum convex medially, without median carina anteriorly; propodeum smooth except with two short carinae latero-posteriorly, with some short setae medially, and densely setose laterally (Fig. 4d).

***Wings***. Fore wing (Fig. 4a): pterostigma slender, 4.4 times longer than wide; SR1: 3-SR: r = 31: 19: 3; 1-SR+M evenly curved; second submarginal cell parallel-sided apically; 2-SR: 3-SR: r-m = 8: 17: 5; m-cu 2.6 times as long as 2-SR+M; cu-a weakly postfurcal. Hind wing (Fig. 4b): 1r-m straight; SC+R1: 2-SC+R: 1r-m = 30: 8: 19.

***Legs***. Length of fore femur: tibia: tarsus = 11: 12: 21; length of hind femur: tibia: basitarsus = 27: 51: 24; length of femur, tibia and basitarsus of hind leg 6.0, 14.6 and 13.3 times their maximum width, respectively; hind tibial spurs 0.2 and 0.3 times as long as hind basitarsus; hind tibia and tarsus densely bristly dark brown setose (Fig. 4f).

***Metasoma***. First tergite relatively slender, 1.9 times longer than its apical width, nearly parallel-sided, median area rather developed, coarsely sculptured (Fig. 4j); first tergite with six long longitudinal rugae on median area, and with developed lateral carinae, lateral grooves narrow (but widened anteriorly) and weakly crenulate (Fig. 4j); apical width of second tergite 1.1 times its median length; triangular medio-basal area of second tergite rather large, apex acute and almost reaching posterior margin of tergite, but not attached to a medio-longitudinal carina, grooves of medio-basal area strongly crenulate, lateral longitudinal grooves deep, widened and crenulate; second tergite coarsely sculptured, largely with longitudinal and oblique striae including medio-basal area (Fig. 4e); second suture rather deep and wide, crenulate, slightly curved upward laterally, nearly straight medially; antero-lateral areas of third tergite convex and distinct; third tergite largely with longitudinal and oblique striae except for posteriorly; antero-lateral areas of fourth tergite rather convex, fourth tergite largely smooth but with longitudinal and oblique striae anteriorly and medially (Fig. 4e); fifth–seventh tergites smooth (Fig. 4e); hypopygium acute apically; ovipositor sheath 2.1 × as long as fore wing.

***Colour***. Head and mesosoma mostly reddish yellow (Fig. 3); antenna largely black, except for scapus yellowish brown with a black longitudinal stripe laterally (Fig. 4i), and apical segments yellowish (Fig. 3); mandible apically black; hind leg and claws black (Fig. 4f); metasomal tergites and ovipositor sheath black (Figs 3, 4e); wing membrane brown, fore wing with irregular pale brown stigmal spot, pterostigma yellowish brown, and veins blackish brown (Figs 4a, b).

##### Distribution.

China (Hainan), Thailand, Malaysia, India.

##### Etymology.

Named after the apico-ventrally protruding scapus: “protuberator” is Latin for “protruding”.

## Disscusion

Typical *Nesaulax* females have both sides of the body conspicuously marked; the apex of the antennal flagellum is pale-tipped – a feature that in Braconidae generally occurs in females and is absent or less distinct in males – and the ovipositor sheath is conspicuously bristly setose and becoming pale near its tip. In general, contrasting colour patterns are associated with species that fly low over leaf litter in moderately dense forests (Quicke 2015). An interesting paper by Quicke et al. (2005) on the behaviour of African *Monilobracon* species (having a white apical plume on the ovipositor sheath) offers a compelling parallel. In *Monilobracon*, the aberrant setosity of the ovipositor sheath is interpreted as a visual signal in territorial defence against other females (Quicke et al. 2005). The “flag-like” display was particularly noticeable against the dark forest background and appeared to be involved in signalling body size or resource-holding potential between conspecific females (Quicke et al. 2005). As pointed out by Quicke (2015), there are other possibilities as being part of a species recognition system, and it may also be confounded by mimicry.

Although the genera are not closely related, and in *Nesaulax* also, the antennae are involved, both conspicuous apical markings may serve a similar role in ritualised intraspecific encounters. The female-specific occurrence of the pale-tipped antenna in *Nesaulax* further supports the idea that such signals are linked to female-specific behaviours such as territorial competition between females. It suggests a potential case of convergent evolution in braconine wasps, in which distinct signalling colouration has been independently selected on two types of body structures to regulate competition among females.

## Supplementary Material

XML Treatment for Nesaulax

XML Treatment for Nesaulax protuberator

## Additional information

### Use of AI

No use of AI was reported.

### Author ORCIDs

Yang Li https://orcid.org/0000-0001-5713-8025

Cornelis van Achterberg https://orcid.org/0000-0002-6495-4853

Jia-Chen Zhu https://orcid.org/0000-0002-1420-5109

Xue-Xin Chen https://orcid.org/0000-0002-9109-8853

### Data availability

All of the data that support the findings of this study are available in the main text or Supplementary Information.

## References

[B1] Enderlein G (1920) Zur Kenntnis aussereuropäischer Braconiden. Archiv für Naturgeschichte 84: 51–224. 10.5962/bhl.part.13627

[B2] Folmer O, Black M, Hoeh W, Lutz R, Vrijenhoek R (1994) DNA primers for amplification of mitochondrial cytochrome c oxidase subunit I from diverse metazoan invertebrates. Molecular Marine Biology and Biotechnology 3: 294–299. 7881515

[B3] Quicke DLJ (1984) Two new genera of Braconinae from the Afrotropical region with a partial review of those genera with ‘merinotoid’ metasomas (Hymenoptera: Braconidae). Entomologist’s Monthly Magazine 120: 37–45.

[B4] Quicke DLJ (2015) The braconid and ichneumonid parasitoid wasps. Biology, Systematics, Evolution and Ecology, [i–xv +] 1–751. [Wiley & Sons, Chichester]. 10.1002/9781118907085

[B5] Quicke DLJ, Laurenne NM, Barclay M (2005) Host, host location and aggressive behaviour in a tropical wood-borer parasitoid genus *Monilobracon* Quicke (Hymenoptera: Braconidae), parasitoids of Lymexylidae (Coleoptera) in Kibale Forest National Park, West Uganda. African Entomology 13(2): 213–220.

[B6] Quicke DLJ, Jasso-Martínez JM, Ranjith AP, Sharkey MJ, Manjunath R, Naik S, Hebert PDN, Priyadarsanan DR, Thurman J, Butcher BA (2023) Phylogeny of the Braconinae (Hymenoptera: Braconidae): A new tribal order! Systematic Entomology 49(1): 84–109 [1–26]. 10.1111/syen.12608

[B7] Roman A (1914) Philippinische Schlupfwespen aus dem schwedischen Reichsmuseum 2. Arkiv för Zoologi 8(24): 1–22. 10.5962/bhl.part.1064

[B8] Taekul C, Valerio AA, Austin AD, Klompen H, Johnson NF (2014) Molecular phylogeny of telenomine egg parasitoids (Hymenoptera: Platygastridae s.l.: Telenominae): evolution of host shifts and implications for classification. Systematic Entomology 39(1): 24–35. 10.1111/syen.12032

[B9] van Achterberg C (1988) Revision of the subfamily Blacinae Foerster (Hymenoptera, Braconidae). Zoologische Verhandelingen Leiden 249: 1–324.

[B10] van Achterberg C (1993) Illustrated key to the subfamilies of the Braconidae (Hymenoptera: Ichneumonoidea). Zoologische Verhandelingen Leiden 283: 1–189.

[B11] Yu DS, van Achterberg C, Horstmann K (2016) Taxapad 2016, Ichneumonoidea 2015. Database on flash-drive. www.taxapad.com [Nepean, Ontario, Canada]

